# Engaging Third-Year Medical Students on Their Internal Medicine Clerkship in Telehealth During COVID-19

**DOI:** 10.7759/cureus.8791

**Published:** 2020-06-24

**Authors:** Heather N Abraham, Ijeoma N Opara, Renee L Dwaihy, Candace Acuff, Brittany Brauer, Renieh Nabaty, Diane L Levine

**Affiliations:** 1 Internal Medicine-Pediatrics, Wayne State University, Detroit Medical Center, Detroit, USA; 2 Internal Medicine, Wayne State University, Detroit Medical Center, Detroit, USA; 3 Medicine, Wayne State University School of Medicine, Detroit, USA

**Keywords:** telehealth, telemedicine, covid-19, ugme, primary care, internal medicine, resident clinic

## Abstract

Purpose: Due to the coronavirus disease-19 (COVID-19) global pandemic, the Association of American Medical Colleges (AAMC) recommended that medical students be removed from contact with patients testing positive or patients under suspicion (PUIs) for COVID-19. As a result of Detroit being a highly affected area, the Wayne State University (WSU) medical students assigned to hospital clerkships during this time were essentially prevented from performing any direct patient care activities. A model for the Internal Medicine (IM) clerkship was developed incorporating a clinical telehealth component, in order to create a safe environment for students to continue to perform meaningful patient care.

Objectives: To model a curriculum whereby students have a diverse patient care experience while increasing their skill and confidence in the performance of telehealth, as measured by self-report in a required pre- and post-clerkship assessment.

Participant population: Twenty, third-year medical students at the end of their academic year, assigned to the IM clerkship at the Detroit Medical Center.

Methods: Students were instructed to complete the American College of Physicians (ACP) module on telehealth, given an orientation via the Zoom online platform by clinical faculty, and placed on a weekly telehealth clinic schedule, precepted by residents and attendings in IM. Survey data was collected covering students’ knowledge, skills, and attitudes surrounding telehealth at the beginning of the rotation. A mid-clerkship feedback session was held with the clerkship director, and the resultant qualitative data was assessed for themes to be compared against the baseline assessment. Determination of incremental change between pre- and post-assessment reports will be evaluated at the completion of the clerkship, with that data forthcoming.

Results: Baseline survey revealed that 90% of students believed the telemedicine experience would be a valuable addition to their IM clerkship. Most were confident that, with training, they could effectively complete a telemedicine visit and 80% felt that telehealth would play an important role in their future careers. Students were pleased with the telemedicine visit logistics and with their role in actively assisting patients with the Zoom online platform. Despite initial anxiety over effectively communicating with patients prior to beginning the telemedicine experience, students demonstrated a common trend towards comfort with that aspect of the visit. Students were impressed with the amount of guidance given by resident and attending physicians in expressing empathy via a virtual platform. Overall, students were pleased with the variety of cases seen and the prompt feedback they received from resident and attending physicians after the telemedicine encounters. At the midpoint assessment, students expressed satisfaction with the overall experience and appreciated the opportunity to continue interacting with patients despite the limitations the pandemic imposed.

Conclusions: Little is formally taught about telehealth in either medical school or medical residency, and integration into a formal curriculum is rare. The AAMC is underway with the development of competencies for telehealth, and, once released, the teaching of this format will become an expectation. We successfully developed a robust model in which medical students not only actively participated in, but also actively delivered, telehealth care to our patients.

## Introduction

The Spring of 2020 has been a time of great anxiety, rapid change, and forced flexibility for medical education due to the coronavirus disease-19 (COVID-19) global pandemic. During this time, the Association of American Medical Colleges (AAMC) recommended that medical student rotations involving care for patients testing positive or patients under suspicion (PUIs) for COVID-19 be paused [1]. As a result of Detroit being a highly affected area, the Wayne State University (WSU) medical students assigned to hospital clerkships during this time were essentially prevented from performing any direct patient care activities. Those on their Internal Medicine (IM) clerkship had their curriculum adjusted to include a clinical telehealth component in order to create a safe environment for continued patient care. The objectives were to provide a diverse patient experience, demonstrate the viability of telehealth in care delivery, and to increase medical student skill and confidence in the performance of telehealth. Despite a resource poor setting, and limitations the pandemic imposed, a meaningful telehealth experience was delivered to the students on the IM clerkship and care was offered to patients who would otherwise have been medically abandoned during this time.

The COVID-19 pandemic forced healthcare infrastructures to rapidly adapt to changes, especially with respect to non-emergent medical care and medical education. As of mid-May 2020, the City of Detroit had a total of 9,973 confirmed cases and 1,218 deaths from the novel coronavirus; the highest of all cities and counties within the state of Michigan [2]. Despite these staggering numbers, medical care and training had to continue uninterrupted. In order to protect patients and healthcare workers, greater focus was placed on service delivery via telehealth.

Prior to the seismic shifts caused by the COVID-19 pandemic, telehealth was considered most beneficial to provide medical care to rural areas and trainee exposure to this practice [3,4]. There had, however, in the years prior to COVID-19 been a growing consensus that all medical students could benefit from telemedicine exposure and training, as seen with support from the American Medical Association [5]. According to AAMC data, by 2017, 60% of medical schools reported including some form of telemedicine training in their curriculum [6]. Medical schools incorporate telemedicine training through various modalities, including lectures, structured clinical experiences, didactics, and electives [7]. There is limited research regarding the methods and impact of telemedicine training in undergraduate medical education, but the consensus is that medical students see benefit in experiencing telemedicine and its positive role in patient care [7,8]. During the COVID-19 global pandemic, the entire spectrum of medical education reworked its method of delivery, with even the United States Medical Licensing Exam (USMLE) accelerating its timeline to telehealthcare assessment via the Step 2 Clinic Skills exam [9]. The WSU IM clerkship educators utilized findings from these limited studies and rapid shifts in skill attainment expectations to quickly and efficiently curate a telemedicine experience for third-year medical students to meet both pandemic and student needs.

## Materials and methods

The 20 students were third years at the end of their academic year, assigned to the IM clerkship at the Detroit Medical Center, and educated by WSU faculty physicians. The students were asked to complete the American College of Physicians (ACP) online learning modules on telemedicine as a foundational introduction to the subject [10]. These modules, made freely available during the COVID-19 pandemic, address “Reasons to Use Virtual Visits, Conducting the Video Visit, Reimbursement, Clinical Use Cases, Success Stories” and take approximately two hours to complete. Students were then engaged via the ZOOM online platform in a 1.5-hour orientation specific to their telehealth rotation, delivered by two clinical faculty members. This orientation focused on the overall concept of telehealth, the model of care delivery via a video platform, the opportunities for patient assessment including performance of a focused visual examination, and the potential benefits to the patient for this modality. Focus was placed on several specific disease states, including hypertension and diabetes, in order to model sample medical assessments. There was a discussion of the significant impact COVID-19 had to date, especially on the underserved and marginalized patients seen in our practice. Emphasis was placed on the importance of evaluating the emotional and social wellbeing of each patient as an integral part of their medical assessment. A Social Determinants of Health (SDH) screening tool was scripted for students to perform during the tele visits, which included community resources to be given to each patient regardless of whether this screen was positive.

The students were assigned to a once-weekly telehealth clinic as a continuity experience, precepted by resident and attending physicians in the IM and Combined Internal Medicine and Pediatrics residency programs. Each medical student was assigned to a schedule of patients, supervised by either both a resident and an attending, or directly supervised by an attending physician. Each schedule had between 6-8 appointments for the half-day session, each with half-hour intervals. The students were asked to initiate the telehealth encounter with each patient on their assigned list. They were asked to do so via Doximity Dialer in order to “mask” their phone number and to ensure that caller identification information came from our resident physician teaching site, the General Medicine and Ambulatory Practice (GMAP) Clinic. Detailed expectations for medical students are discussed in Table 1.

**Table 1:** Expectations for medical students performing telehealth in GMAP during COVID-19 *GMAP clinic staff performed a ZOOM trial run with patients the day or two prior to the appointment. This was limited, however, as staff members were actively furloughed during our adaptation of this workflow. Medical students, therefore, became an even more integral part of a smooth telehealth delivery process. GMAP: General Medicine and Ambulatory Practice; HIPAA: Health Insurance Portability and Accountability Act

Encounter Set-up	Professional attire, including white coat if available
	Secure/private/quite location for encounter, HIPAA compliant
	Neutral background, home office, etc. or generic green screen; NOT bedroom
	Prepared about 30 minutes prior to first appointment time
	EMR access, phone with Doximity Dialer, etc.
Appointment Initiation	Call patient via Doximity Dialer with GMAP caller ID
(10 minutes prior to appointment start time)	Introduce self, remind patient of appointment scheduled in 10 mins
	Confirm patient will be in safe/secure/private location for duration of visit
	Transition patient to ZOOM, give meeting codes from email sent by physician
	Troubleshoot patient logging onto the ZOOM online platform*
	Perform scripted Social Determinants of Health screening
	Introduce physician joining the call at the appointed time
During Encounter	Remotely “shadow” the history and physical performed by the physician
	Physicians will escalate the responsibilities of the students as comfort level is achieved with goal of student initiation, performance, and televisit wrap-up
Post Encounter	Patient exits encounter, physicians and student remain online
	Feedback given in real time to medical students and trainees via ZOOM

These students participated in this clinic from mid-April 2020 through the end of May 2020, encompassing the greatest peak of COVID-19 cases in Detroit. Published studies to date regarding telehealth training for medical students had commonly used pre- and post-tests or surveys, in addition to qualitative feedback, as a means to track student interest in and skill with telehealth [3,11]. As such, a pre-assessment based on these studies was designed, distributed as an email, and collected through WSU Qualtrics. The pre-assessment covered reports of familiarity with telehealth in general, comfort level with delivering quality patient care via this modality, including adequate assessment of social concerns, and importance placed on teaching telehealth as part of undergraduate medical education. A post-assessment will evaluate changes in these parameters, as well as capture details of feedback methods employed by educators.

## Results

Baseline survey data

Fifteen of the 20 assigned students (75%) participated in the pre-assessment and only ten reported having completed the ACP module (Table 2). Only two respondents were more than “moderately familiar” with the concept of telehealth, however, all but one acknowledged that learning about it would be valuable to their clinical education. In a similar question, more than 90% agreed that incorporating telehealth into the IM clerkship would improve the experience, and 80% signified that telehealth would become an important part of their future career in medicine. The pre-clerkship assessment can be viewed in the appendices.

**Table 2:** Pre-clerkship assessment survey data

	(n=15)	(n=15)	(n=15)	(n=15)	(n=15)
	Strongly agree	Somewhat agree	Neutral /Neither	Somewhat disagree	Strongly disagree
Telehealth is a valuable component to my clinical education.	53%	40%	0%	7%	0%
Telehealth is a valuable service to offer to patients.	87%	13%	0%	0%	0%
I am comfortable with the ZOOM platform.	47%	47%	6%	0%	0%
I am confident in my ability to perform a limited physical exam on a patient via telehealth.	7%	33%	53%	0%	7%
The script I was given to assess for my patients' social needs is helpful	53%	40%	7%	0%	0%
Doctors can provide quality care to their patients via telehealth	53%	40%	0%	7%	0%
Incorporating a telehealth learning opportunity into my IM clerkship improves my clerkship experience.	60%	33%	0%	7%	0%
Telehealth will be an important part of my future career in medicine.	40%	40%	13%	7%	0%
	Extremely familiar	Very familiar	Moderately familiar	Slightly familiar	Not at all
I am familiar with the concept of telehealth.	0%	14%	57%	21%	7%
	Extremely comfortable	Somewhat comfortable	Neither	Somewhat uncomfortable	Extremely uncomfortable
I am comfortable completing a telehealth visit with a patient.	7%	60%	20%	13%	0%
I believe my patients will be comfortable completing telehealth visits with their doctor.	7%	80%	13%	0%	0%
	Definitely yes	Probably yes	Might or might not	Probably not	Definitely not
I am confident that I can assess for social needs and social determinants of health on a telehealth visit.	43%	57%	0%	0%	0%
	Extremely adequate	Somewhat adequate	Neither	Somewhat inadequate	Extremely inadequate
The resource list I was given is adequate.	20%	67%	13%	0%	0%

In general, respondents were familiar with ZOOM, however, there was a wide range of comfort reported with completing a telehealth visit with a patient via the platform, with the most common response being only “somewhat comfortable.” Half were neutral with respect to confidence performing a limited physical exam on a virtual visit. All respondents were “probably” or “definitely” confident in their ability to assess social needs and social determinants of health on a telehealth visit, with most in agreement that the SDH screening tool (see appendices) and resource list were useful.

All respondents agreed that telehealth is a valuable service to offer patients, and none felt patients would be uncomfortable completing this type of visit with their doctor. Ninety-three percent of respondents agreed that doctors can provide quality care to their patients over telehealth.

Mid-clerkship evaluation summary

In May 2020, 18 out of the 20 students (90%) met with the IM clerkship director to provide feedback on their telehealth experience to date, serving as a mid-clerkship assessment. Student feedback was synthesized in a qualitative analysis and tagged to five topics: logistics, communication skills, case mix exposure, feedback, and their overall telehealth experience.

*Logistics*: A common theme was satisfaction with the logistics of their telehealth experience. All students reported satisfaction with calling patients and providing education regarding the ZOOM platform. Although many students reported that patients faced challenges with technology, the students viewed this as an opportunity to educate and assist patients, and had no difficulty providing instruction and guidance. No students reported issues with personal access to technology, including reliable Wi-Fi.

*Communication Skills*: Medical students are trained to take a full history, utilize superior communication skills, and practice empathy during a clinical encounter with patients. Prior to engaging in the telehealth curriculum, students reported anxiety surrounding the ability to develop a rapport with patients during televisits. One student continued to report having trouble with communicating empathy during a telehealth visit at the mid-clerkship. During what was described as an “impactful” encounter, the student was able to receive real-time guidance and feedback on how to communicate and practice empathy when conducting telehealth visits, from the attending physician who was able to demonstrate this skill on the call. Students also reported satisfaction with utilizing the scripted SDH screening tool and enjoyed engaging with the patients in the screening process.

*Case Mix Exposure*: Students reported being pleasantly surprised by the variety of cases they saw during their telehealth experience. They reported a wide variety of visit reasons, including COVID-19 follow ups, evaluation of acute care concerns like rashes and red eyes, management of chronic conditions from diabetes and hypertension to bedsores and mental health diagnoses, engaging guardians in caring for patients with disabilities like cerebral palsy and autism, and refilling medications.

*Feedback*: Students expressed appreciation of timely feedback during telehealth encounters. Some reported that feedback sessions occurred immediately after the televisit concluded, with the resident or attending physician staying “on the line” to provide feedback once the visit ended and the patient was no longer virtually present.

*Overall Experience*: At the time of the mid-point evaluation, students reported being highly satisfied with their telehealth experience. Students reported that everything was going well and described the experience as a “great, pretty cool, and interesting” opportunity. Students also reported being pleased that they had been able to continue to have contact with patients, while adhering to AAMC and governmental social distancing requirements.

## Discussion

Time was of the essence during COVID-19, and innovation was spurred by necessity. The duration from inception of this project and first approach to the Vice Chair for Medical Education, to the students’ first telehealth session was exactly one week. In that compressed timeframe, the lead faculty were able to leverage available resources, including the significant support of the educational leadership, in order to integrate medical students into the clinical telehealth plan. In less than a week, in addition to preparing physicians and staff for telehealth visits, the student orientation was developed and delivered, the schedule was created, and the pre-assessment survey was structured.

Based on the pre-assessment, students were enthusiastic about telehealth; they placed importance on offering this to patients and learning the model. Most students, however, were not confident in their ability to deliver a high-quality visit nor to perform a limited physical exam via only synchronous audio/video or telephone. Examination of student reports from their mid-clerkship conversation already demonstrate an obvious shift from that baseline pre-assessment.

Formal assessment of skilled telehealth delivery was difficult, given the limited training all practitioners had to date prior to the COVID-19 emergency. Feedback was given in a visible, yet less structured way than may otherwise be when evaluating more common skills, physical exam, etc. A checklist, such as that proposed by Jonas et al. could be a valuable tool to standardize the evaluation of telehealth, and to make uniform the quality of each encounter [11]. The post-assessment will further characterize feedback structure and medical student satisfaction with its quality, and may give further support for telehealth as a way to more actively engage learners and educators.

Moving forward, integration of this program into the fabric of the clerkship and the medical school experience will be paramount. This is technology that will not slink back into obscurity. Formal milestones and competencies from the AAMC are reported to be forthcoming, and, when available, will be incorporated into future iterations of this clerkship model. Failure to sustain this momentum is a possibility, especially once the relaxation of restrictions surrounding telehealth delivery and billing changes rolled out during COVID-19 are, at least temporarily, rolled back once the pandemic emergency is resolved. Once payment parity for telehealth encounters with in-office visits is no longer guaranteed, administrative enthusiasm for telehealth will likely wane. This program will thrive only with the continued support of academic and clinical leadership, as well as with the energy of faculty champions.

The success of the clerkship described here is an opportunity on which to capitalize. Other data that may bolster the case to continue with telehealth is the exploration of patient satisfaction with the model, and the potential that offering this might make a patient choose one provider or group over another. COVID-19 has irrevocably changed the world and the practice of medicine. An integrated practice model, with primary care being delivered via a combined brick and mortar medical home as well as via telehealth, may no longer be optional, but necessary.

## Conclusions

Incorporating telemedicine into the IM clerkship provided our junior students with a meaningful clinical experience during a time when in person patient care was otherwise deemed unsafe. Comparison between students’ self-reported familiarity and comfort with telehealth delivery during a clerkship pre-assessment and a mid-clerkship evaluation demonstrated significant improvements. Most notably, students seemed to feel increasingly confident in their ability to build rapport with patients after performing several telehealth visits, assuaging a commonly cited concern among physicians unwilling to adopt telehealth for their practice. Case mix was broad, encompassing not only the requisite COVID-19 follow up or diagnosis, but also chronic disease management and mental health issues. Students found that numerous patient concerns could be addressed via telehealth, a fact that proponents of the modality will also champion. Student satisfaction with the curriculum was high, and nearly half of students reached out to course directors or clinic attendings seeking to add additional sessions. Despite myriad challenges, resource limitations, anxiety about telehealth, and low confidence in our patients, physicians and learners to rapidly adapt to change, we successfully developed a model in which medical students not only actively participated in, but also actively delivered, telehealthcare to our patients.

## Appendices

Pre-clerkship assessment

Q1 I am familiar with the concept of telehealth.

o Extremely familiar  (1)

o Very familiar  (2)

o Moderately familiar  (3)

o Slightly familiar  (4)

o Not familiar at all  (5)

Q2 Telehealth is a valuable component of my clinical education.

o Strongly agree  (1)

o Somewhat agree  (2)

o Neither agree nor disagree  (3)

o Somewhat disagree  (4)

o Strongly disagree  (5)

Q3 Telehealth is a valuable service to offer to patients.

o Strongly agree  (1)

o Somewhat agree  (2)

o Neither agree nor disagree  (3)

o Somewhat disagree  (4)

o Strongly disagree  (5)

Q4 I completed the ACP Telehealth module.

o Yes  (1)

o No  (2)

Q5 I am comfortable with the ZOOM platform.

o Strongly agree  (1)

o Somewhat agree  (2)

o Neither agree nor disagree  (3)

o Somewhat disagree  (4)

o Strongly disagree  (5)

Q6 I am comfortable completing a telehealth visit with a patient.

o Extremely comfortable  (1)

o Somewhat comfortable  (2)

o Neither comfortable nor uncomfortable  (3)

o Somewhat uncomfortable  (4)

o Extremely uncomfortable  (5)

Q7 I believe my patients will be comfortable completing telehealth visits with their doctor.

o Extremely comfortable  (1)

o Somewhat comfortable  (2)

o Neither comfortable nor uncomfortable  (3)

o Somewhat uncomfortable  (4)

o Extremely uncomfortable  (5)

Q8 I am confident in my ability to perform a limited physical exam on a patient via telehealth.

o Strongly agree  (1)

o Somewhat agree  (2)

o Neither agree nor disagree  (3)

o Somewhat disagree  (4)

o Strongly disagree  (5)

Q9 I am confident that I can assess for social needs and social determinants of health on a telehealth visit.

o Definitely yes  (1)

o Probably yes  (2)

o Might or might not  (3)

o Probably not  (4)

o Definitely not  (5)

Q10 The script I was given to assess for my patients' social needs is helpful.

o Strongly agree  (1)

o Somewhat agree  (2)

o Neither agree nor disagree  (3)

o Somewhat disagree  (4)

o Strongly disagree  (5)

Q11 The resource list I was given is adequate.

o Extremely adequate  (1)

o Somewhat adequate  (2)

o Neither adequate nor inadequate  (3)

o Somewhat inadequate  (4)

o Extremely inadequate  (5)

Q12 Doctors can provide quality care to their patients via telehealth.

o Strongly agree  (1)

o Somewhat agree  (2)

o Neither agree nor disagree  (3)

o Somewhat disagree  (4)

o Strongly disagree  (5)

Q13 Incorporating a telehealth learning opportunity into my Internal Medicine clerkship improves my clerkship experience. 

o Strongly agree  (1)

o Somewhat agree  (2)

o Neither agree nor disagree  (3)

o Somewhat disagree  (4)

o Strongly disagree  (5)

Q14 Telehealth will be an important part of my future career in medicine.

o Strongly agree  (1)

o Somewhat agree  (2)

o Neither agree nor disagree  (3)

o Somewhat disagree  (4)

o Strongly disagree  (5)

Q15 Please add any additional comments you have about telehealth.

________________________________________________________________

Brief screening for social needs, aka “SDH Screening Tool”

STUDENT: "Good day, Mr/Ms ________. My name is Dr. ____________ How are you today?  We understand that the CoVID-19 pandemic is affecting our community in many ways.  As we work to make sure our patients' healthcare needs are met during this challenging time, we also want to ensure that your social needs are not been overlooked because we know that they have a great impact on your overall health. Are you having any difficulty with paying bills, access to food, clothing, housing, obtaining your medication, mental health, transportation, childcare, or any such concern?"

Scenario 1:

PATIENT: "NO."

STUDENT: "GREAT! So glad to hear that! If any such concern arises in the future, though, please call 211, the community essential services hotline.

Scenario 2: 

PATIENT: "YES" (proceeds to share what their concerns are) 

STUDENT: "I'm so sorry to hear that. Many people are struggling with these concerns as well. Please call the 211 number, which is the community essential service number. Or you can visit their website, www.mi211.org. Someone will be able to help you with this."

If you encounter a concern that you are unable to address, take their number and staff will workshop it through and provide follow up. 

Specific Social Needs Resources:

https://www.mi211.org/

https://www.navigateresources.net/uwse/

https://www.auntbertha.com/

http://julieslist.homestead.com/?fbclid=IwAR1GO3NM1GwtYt1iU9ZQTnznuP2hb9z1TUGrusHBBeFLLwOyPYkOe5MHRG4

https://www.facebook.com/Julies-List-Website-128098500591417/

Interactive map for where children can get meals, neighborhood mutual aid, water drop off, etc. 

https://www.bridgemi.com/michigan-health-watch/michigan-families-can-get-food-cash-internet-during-coronavirus-crisis

## References

[1] (2020). Association of American Medical Colleges: updated interim guidance for medical students’ participation in patient care during the coronavirus (COVID-19) outbreak. https://www.aamc.org/news-insights/press-releases/updated-interim-guidance-medical-students-participation-patient-care-during-coronavirus-covid-19.

[2] (2020). Michigan coronavirus data. https://www.michigan.gov/coronavirus/0,9753,7-406-98163_98173---,00.html.

[3] Bonney A, Knight-Billington P, Mullan J (2015). The telehealth skills, training, and implementation project: an evaluation protocol. JMIR Res Protoc.

[4] Wernhart A, Gahbauer S, Haluza D (2019). eHealth and telemedicine: practices and beliefs among healthcare professionals and medical students at a medical university. PLoS One.

[5] (2020). American Medical Association encourages telemedicine training for medical students, residents. https://www.ama-assn.org/press-center/press-releases/ama-encourages-telemedicine-training-medical-students-residents.

[6] Pourmand A, Ghassemi M, Sumon K, Amini SB, Hood C, Sikka N (2020). Lack of telemedicine training in academic medicine: are we preparing the next generation? [Epub ahead of print]. Telemed J E Health.

[7] Waseh S, Dicker AP (2019). Telemedicine training in undergraduate medical education: mixed-methods review. JMIR Med Educ.

[8] Pathipati AS, Azad TD, Jethwani K (2016). Telemedical education: training digital natives in telemedicine. J Med Internet Res.

[9] (2020). United States Medical Licensing Examination update on resuming testing. https://www.usmle.org/announcements/.

[10] (2020). American College of Physicians: telemedicine: a practical guide for incorporation into your practice. https://www.acponline.org/cme-moc/online-learning-center/telemedicine-a-practical-guide-for-incorporation-into-your-practice.

[11] Jonas CE, Durning SJ, Zebrowski C, Cimino F (2019). An interdisciplinary, multi-institution telehealth course for third-year medical students. Acad Med.

